# Polynucleotides Enhance Collagen Synthesis via Modulating Phosphoenolpyruvate Carboxykinase 1 in Senescent Macrophages: Experimental Evidence

**DOI:** 10.3390/ijms26178720

**Published:** 2025-09-07

**Authors:** Kyung-A Byun, Hyun Jun Park, Seyeon Oh, Kuk Hui Son, Kyunghee Byun

**Affiliations:** 1 Department of Anatomy & Cell Biology, College of Medicine, Gachon University, Incheon 21936, Republic of Korea; 2 LIBON Inc., Incheon 22006, Republic of Korea; 3 Maylin Clinic Cheongdam, Seoul 06015, Republic of Korea; 4 Functional Cellular Networks Laboratory, Lee Gil Ya Cancer and Diabetes Institute, Gachon University, Incheon 21999, Republic of Korea; 5 Department of Thoracic and Cardiovascular Surgery, Gachon University Gil Medical Center, Gachon University, Incheon 21565, Republic of Korea; 6 Department of Health Sciences and Technology, Gachon Advanced Institute for Health & Sciences and Technology (GAIHST), Gachon University, Incheon 21999, Republic of Korea

**Keywords:** adenosine 2A receptor, collagen synthesis, phosphoenolpyruvate carboxykinase 1, polynucleotides, rejuvenation

## Abstract

Polynucleotide (PN), a high-molecular-weight DNA fragment derived from salmon and other fish sources, shows promising anti-aging and regenerative effects on the skin. This study investigated how PN enhances collagen synthesis, focusing on its effect on phosphoenolpyruvate carboxykinase 1 (PCK1) in senescent macrophages and its downstream effects on fibroblasts. Using in vitro senescent cell models and in vivo aged animal models, PN significantly upregulated the adenosine 2A receptor (A2AR), adenylate cyclase (AC), cyclic AMP (cAMP), protein kinase A (PKA), and cAMP response element-binding protein (CREB) in senescent macrophages. This led to increased PCK1 expression, which reduced oxidative stress and promoted M2 macrophage polarization, associated with elevated levels of interleukin-10 and tumor growth factor-β. Conditioned media from PN-treated macrophages enhanced SMAD family member 2 and signal transducer and activator of transcription 3 phosphorylation in senescent fibroblasts, increasing collagen I and III synthesis and reducing nuclear factor-κB activity. In vivo, PN administration elevated expression of the A2AR/AC/PKA/CREB/PCK1 pathway, reduced oxidative stress, increased M2 macrophage markers, and significantly improved collagen density and skin elasticity over time. Use of a PCK1 inhibitor attenuated these effects, highlighting the pivotal role of PCK1. Overall, PN modulates macrophage-fibroblast interactions via the CREB/PCK1 axis, enhancing collagen synthesis and counteracting age-related skin changes. PN has emerged as a promising therapeutic agent for skin rejuvenation by targeting cellular senescence and promoting extracellular matrix restoration.

## 1. Introduction

Polydeoxyribonucleotide (PDRN) is a DNA fragment with a molecular weight ranging from 50 to 1500 kDa and is extracted from the sperm of salmon trout or chum salmon [1]. Polynucleotides (PNs) have large molecular weights (<1500 kDa) and can be extracted from the testis of Pacific salmon, sturgeons, and rainbow trout [2,3].

PDRN exerts anti-inflammatory effects by stimulating the adenosine 2A receptor (A2AR) [4]. It is degraded into nucleotides by endogenous nucleases. These nucleotides bind to A2AR, which upregulates adenylate cyclase (AC) and subsequently increases cyclic AMP (cAMP) [1,5]. Increased cAMP upregulates protein kinase A (PKA), which increases cAMP response element-binding protein (CREB) levels and decreases MAPK pathways, subsequently decreasing inflammation [6]. PDRN also decreased nuclear factor (NF)-κB phosphorylation, leading to decreased tumor necrosis factor (TNF)-α and interleukin (IL)-1β expression by PKA/CREB upregulation [7]. By decreasing inflammation, PDRN stimulates wound healing and inhibits the degradation of extracellular matrix (ECM) such as collagen [8]. Moreover, PDRN increases the levels of anti-inflammatory cytokines such as IL-10 [9].

CREB activation leads to the upregulation of phosphoenolpyruvate carboxykinase 1 (PCK1), a key rate-limiting enzyme in gluconeogenesis [10,11]. PCK1 also induced changes in oxidative stress. Upon overexpression of PCK1, nicotinamide adenine dinucleotide phosphate (NADPH), which inhibits reactive oxygen species (ROS) generation, increased [12]. In contrast, the deletion of PCK1 led to increased ROS, which is associated with increased proinflammatory-type macrophages (M1) [13]. Unlike M1 macrophages, M2 macrophages secrete anti-inflammatory cytokines such as IL-10 and tumor growth factor (TGF)-β, which stimulates tissue repair [14].

TGF-β increases the synthesis of various ECM materials, such as collagen, via phosphorylation of SMAD family member 2 (SMAD2) [15]. IL-10 decreased inflammation by inhibiting NF-κB [16]. Moreover, IL-10 upregulates cytoplasmic receptor-associated tyrosine kinases, such as janus kinase 1 and tyrosine kinase 2, which leads to the phosphorylation of signal transducer and activator of transcription 3 (STAT3) [17]. IL-10 also inhibited NF-κB via STAT3 [18]. NF-κB increased matrix metalloproteinase (MMP)-1 and -3, which induced ECM destruction [19]. Thus, elevated IL-10 levels may protect ECM by inhibiting downstream NF-κB signaling.

With aging, the oxidative stress in the skin increases and is associated with increased chronic inflammation [20]. NF-κB activity and the expression of MMPs are increased in the aged tissue [20]. In aged animal skin, M2 macrophages and IL-10 levels are significantly reduced compared to young skin [21]. These changes lead to decreased collagen synthesis and increased collagen destruction, resulting in age-related changes in the skin, such as wrinkles, sagging, and loss of facial volume [22].

Given its well-documented anti-inflammatory properties and capacity to enhance collagen synthesis, PDRN has emerged as a promising anti-aging compound capable of counteracting key pathological mechanisms observed in aging skin [23]. The close molecular resemblance between PN and PDRN suggests that their mechanisms of action may be similar [24].

Building on previous evidence that PDRN activates adenosine receptors to exert anti-inflammatory effects [4,7], we hypothesized that PN enhances collagen synthesis by regulating PCK1 expression. We proposed that PN activates the A2AR to stimulate the downstream CREB/PCK1 pathway in senescent macrophages. This activation leads to a reduction in oxidative stress and promotes M2 polarization. We further hypothesized that M2-polarized macrophages subsequently promote fibroblasts to enhance collagen synthesis.

To investigate this hypothesis, we evaluated whether PN affects PCK1 to induce M2 polarization. Moreover, we evaluated whether M2-secreted IL-10 and TGF-β could enhance STAT3 and SMAD2, respectively, leading to reduced collagen degradation through STAT3-mediated inhibition of NF-κB and increased collagen synthesis via SMAD2 activation, using both in vitro senescent cell models and in vivo aged animal models.

## 2. Results

### 2.1. PN Increased A2AR, CREB, and PCK1 in the Senescent Macrophage

A senescent macrophage model was generated by treating cells with hydrogen peroxide (H_2_O_2_). The senescent phenotype was confirmed by increased expression of senescent markers p16 and p21 (Figure S1).

**Figure 1:**
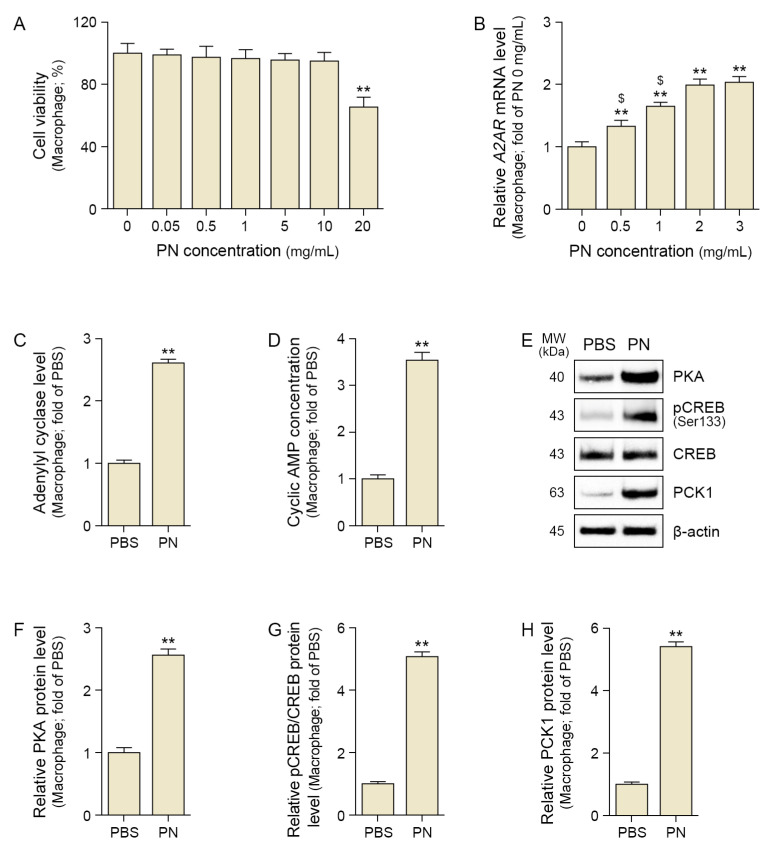
PN activates the A2AR–cAMP–PKA/CREB pathway in senescent macrophages. (**A**) Cytotoxicity of PN was assessed using CCK-8 assay. (**B**) Quantitative polymerase chain reaction analysis of A2AR expression in response to PN. (**C**) Adenylate cyclase level was elevated after PN treatment using assay kit. (**D**) Cyclic AMP levels increased in PN-treated macrophages using assay kit. (**E**–**H**) Western blot analysis revealed increased expression of PKA (**F**), phosphorylated CREB (pCREB, Ser133), CREB (**G**), and PCK1 (**H**) following PN treatment. β-actin served as the loading control. (**F**–**H**) Densitometric quantification of the Western blot bands shown in (**E**). Data are expressed as the mean ± SD. **, *p* < 0.01, vs. first bar; $, *p* < 0.05, vs. fourth bar (Mann–Whitney U test). A2AR, adenosine 2A receptor; CREB, element-binding protein; MW, molecular weight; PBS, phosphate-buffered saline; PCK1, phosphoenolpyruvate carboxykinase 1; PKA, protein kinase A; PN, polynucleotides.

The PN concentration that did not affect cell viability was determined. PN at 10 mg/mL did not affect the viability of senescent macrophages (Figure 1A).

The optimal concentration of PN for the in vitro experiments was determined based on its effect on A2AR. Treatment with PN at a concentration of 0.5 mg/mL began to increase A2AR expression, and this expression continued to rise as the PN concentration increased. However, there was no significant difference in A2AR expression between the 2 mg/mL and 3 mg/mL PN concentrations, suggesting a saturation effect. Based on these results, we determined that 2 mg/mL PN is the most effective concentration for increasing A2AR. To simplify subsequent experiments, we proceeded by using only the 2 mg/mL PN concentration. (Figure 1B).

PN significantly increased the expression of AC and cAMP in senescent macrophages (Figure 1D). PN also increased PKA, the ratio of phosphorylated CREB to total CREB (pCREB/CREB), and PCK1 in senescent macrophages (Figure 1E–H).

### 2.2. PN Decreased Oxidative Stress and Increased M2 Polarization in the Senescent Macrophages

To determine whether PN-induced stimulation of PCK1 mitigates oxidative stress and enhances M2 polarization in senescent macrophages, cells were treated with a PCK1 inhibitor (3-MPA; 3-mercaptopicolinic acid hydrochloride) and compared with phosphate-buffered saline (PBS)-treated controls (Figure S2).

**Figure 2:**
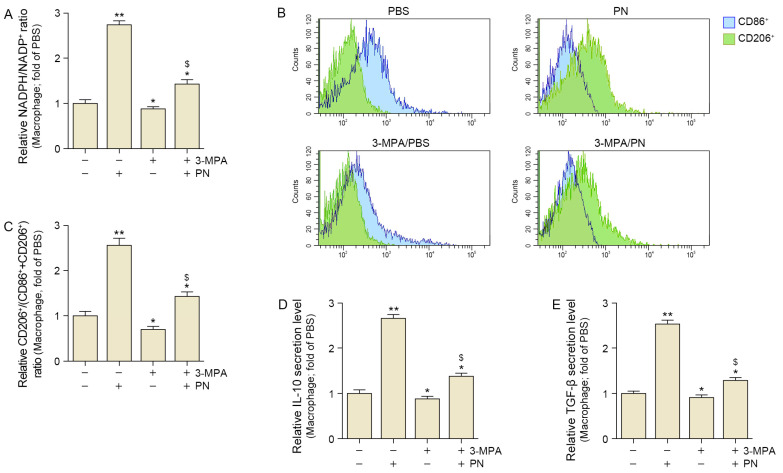
PN regulated oxidative stress and M2 polarization in phosphoenolpyruvate carboxykinase 1 (PCK1)-dependent senescent macrophages. (**A**) Relative NADPH/NADP^+^ ratio was significantly increased after PN treatment, and this effect was suppressed by 3-MPA in senescent macrophages. (**B**,**C**) Representative flow cytometry histograms showing surface marker expression of CD86^+^ and CD206^+^ macrophages under the indicated treatments (PBS, PN, with or without 3-MPA). (**D**,**E**) ELISA results show increased secretion of anti-inflammatory cytokines IL-10 (**D**) and TGF-β (**E**) after PN treatment, and this effect was suppressed by 3-MPA in senescent macrophages. The (+) and (−) symbols indicate the presence (+) or absence (−) of the indicated treatment (PN or 3-MPA). Data are expressed as the mean ± SD. *, *p* < 0.05 and **, *p* < 0.01, vs. first bar; $, *p* < 0.05, third bar vs. fourth bar (Mann–Whitney U test). 3-MPA, 3-mercaptopicolinic acid hydrochloride; NADPH, nicotinamide adenine dinucleotide phosphate; IL-10, interleukin-10; PBS, phosphate-buffered saline; PN, polynucleotides; TGF-β, tumor growth factor-β.

Oxidative stress was evaluated using NADPH/NADP^+^ which is a frequently used marker of oxidative stress [25]. PN treatment increased the NADPH/NADP^+^ ratio, indicating reduced oxidative stress in senescent macrophages. However, 3-MPA decreased the PN-induced increase in NADPH/NADP^+^. These results suggest that PN decreases oxidative stress via PCK1 (Figure 2A).

Flow cytometry was used to quantify the populations of M1 and M2 macrophages by identifying CD86^+^ cells and CD206^+^ cells, respectively. To determine the extent of M2 polarization, we used a ratio calculated by dividing the number of CD206^+^ cells by the total number of both CD86^+^ and CD206^+^ cells. PN increased CD206^+^ cell/(CD86^+^ cell + CD 206^+^ cell), but these increases were attenuated by 3-MPA in the senescent macrophages (Figure 2B,C).

Additionally, PN increased IL-10 and TGF-β levels, but these increases were attenuated by 3-MPA in the senescent macrophages (Figure 2D,E).

These results suggest that PN increased M2 polarization and secretion of M2 cytokines such as IL-10 and TGF-β via PCK1.

### 2.3. PN Increased SMAD2/3 and STAT3 Which Increased Collagen in the Fibroblasts

Since we hypothesized that PN affects macrophages, which in turn influence fibroblasts to induce collagen synthesis, we treated fibroblasts with conditioned media (CM) from senescent macrophages. Senescent fibroblasts were generated using H_2_O_2,_ and the expression levels of p16 and p21 were evaluated (Figure S3).

**Figure 3:**
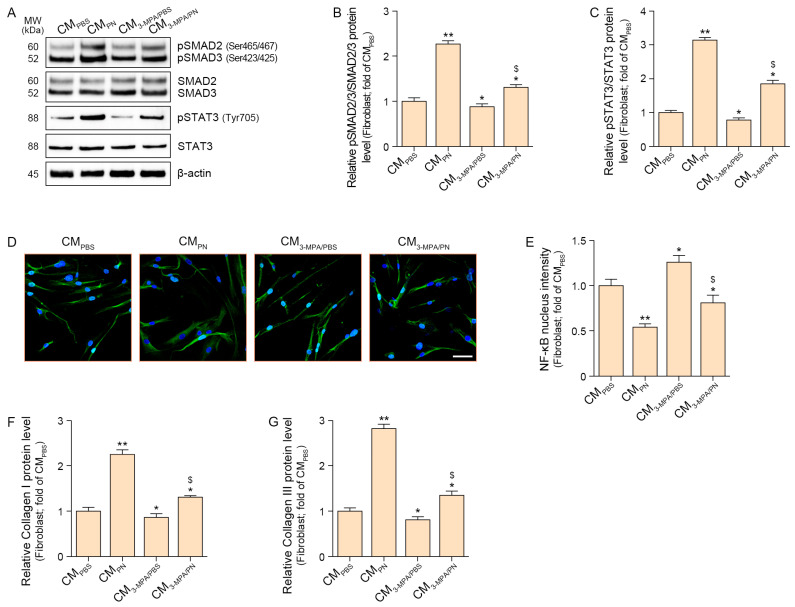
PN regulated increased collagen in PCK1-dependent senescent fibroblasts. (**A**–**C**) Western blot analysis showed that the expression levels of phosphorylated SMAD2/3 (pSMAD2 Ser465/467, pSMAD3 Ser423/425; (**B**) and STAT3 (pSTAT3 Tyr705; (**C**) were increased. β-actin served as the loading control. (**B**,**C**) Densitometric quantification of the Western blot bands shown in (**A**). (**D**) Representative immunocytochemistry images for NF-κB localization in senescent fibroblasts (scale bar = 50 μm). (**E**) Quantification of nuclear NF-κB translocation from (**D**). (**F**,**G**) ELISA analysis showing changes in Collagen I (**F**) and Collagen III (**G**) protein levels in senescent fibroblasts treated with macrophage supernatants. Data are expressed as the mean ± SD. *, *p* < 0.05 and **, *p* < 0.01, vs. first bar; $, *p* < 0.05, third bar vs. fourth bar (Mann–Whitney U test). 3-MPA, 3-mercaptopicolinic acid hydrochloride; CM, conditioned media; PBS, phosphate-buffered saline; PN, polynucleotides.

The ratio of pSMAD2/3 to total SMAD2/3 (pSMAD2/3/SMAD2/3) was increased by the CM from PN-treated senescent macrophages (CM_PN_) to senescent fibroblasts. This increase was mitigated when fibroblasts were treated with CM from PN- and 3-MPA-treated senescent macrophages (CM_3-MPA/PN_) (Figure 3A,B).

The ratio of pSTAT3 to total STAT3 (pSTAT3/STAT3) was increased by CM_PN_ in the senescent fibroblasts. This increase was mitigated by CM_3-MPA/PN_ (Figure 3A,C).

NF-κB activation was evaluated by measuring its nuclear intensity, which was decreased by CM_PN_ in senescent fibroblasts. This decrease was mitigated by CM_3-MPA/PN_ (Figure 3D,E).

Expression of collagen I and III was increased by CM_PN_ in senescent fibroblasts. This decrease was mitigated by CM_3-MPA/PN_ (Figure 3F,G).

These results suggest that PN increases collagen levels in senescent fibroblasts by modulating macrophage PCK1.

### 2.4. PN Increased A2AR, AC, PKA, CREB, and PCK1 in the Aged Skin

**Figure 4:**
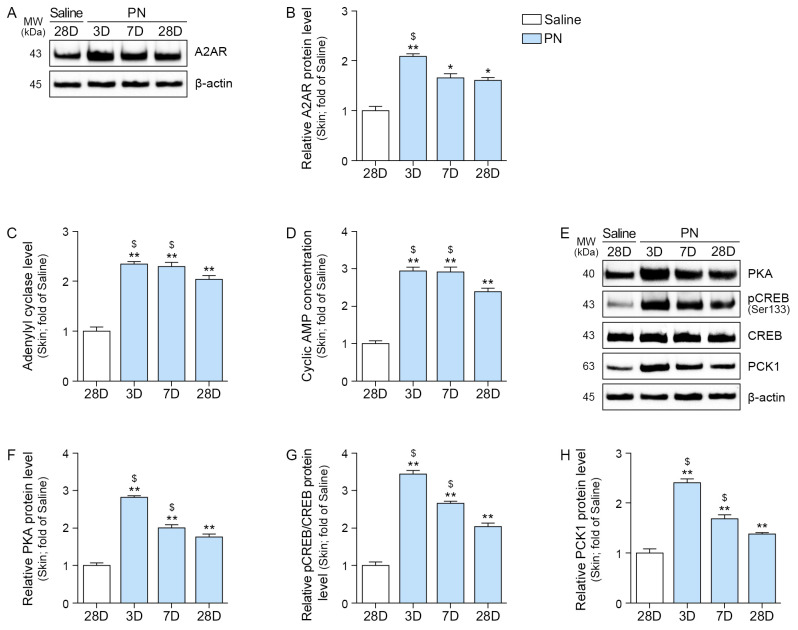
PN activates the A2AR–cAMP–PKA/CREB signaling pathway in mouse skin. (**A**,**B**) Western blot analysis of A2AR protein levels in mouse skin at 3, 7, and 28 days after PN treatment. β-actin served as the loading control. (**B**) Quantification of A2AR expression from (**A**). (**C**) Adenylate cyclase level in mouse skin following PN treatment. (**D**) Cyclic AMP levels in mouse skin following PN treatment. (**E**–**H**) Western blot analysis of expression of PKA (**F**), phosphorylated CREB (pCREB, Ser133), CREB (**G**), and PCK1 (**H**) in mouse skin at 3, 7, and 28 days after PN treatment. β-actin served as the loading control. (**F**–**H**) Densitometric quantification of the Western blot bands shown in (**E**). Data are expressed as the mean ± SD. *, *p* < 0.05 and **, *p* < 0.01, vs. first bar; $, *p* < 0.05, vs. fourth bar (Mann–Whitney U test). A2AR, adenosine 2A receptor; CREB, element-binding protein; MW, molecular weight; PCK1, phosphoenolpyruvate carboxykinase 1; PKA, protein kinase A; PN, polynucleotides.

PN increased the expression of A2AR, AC, and cAMP in aged skin (Figure 4A–D). Moreover, PN increased PKA, pCREB/CREB, and PCK1 levels in aged skin (Figure 4E–H). This increase was the most prominent three days after treatment with PN.

### 2.5. PN Decreased Oxidative Stress and Increased M2, IL-10, TGF-β, SMAD2/3, and STAT3 in the Aged Skin

**Figure 5:**
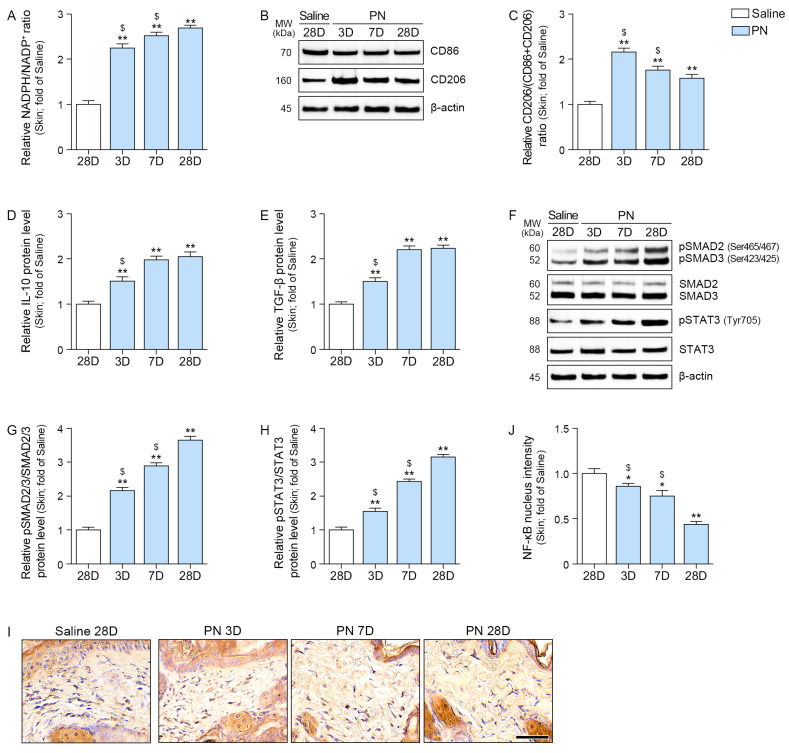
PN regulated oxidative stress, M2 polarization, anti-inflammatory cytokines, SMAD2/3, and STAT3 in mouse skin. (**A**) Relative NADPH/NADP^+^ ratio was significantly increased following PN treatment. (**B**,**C**) Western blot analysis of CD86 and CD206 expression in mouse skin at 3, 7, and 28 days after PN treatment. β-actin served as the loading control. (**C**) Quantification of CD206/(CD86+CD206) expression from (**B**). (**D**,**E**) ELISA results show increased expression of anti-inflammatory cytokines IL-10 (**D**) and TGF-β (**E**) after PN treatment. (**F**–**H**) Western blot analysis showing that the expression levels of phosphorylated SMAD2/3 (pSMAD2 Ser465/467, pSMAD3 Ser423/425; (**G**) and STAT3 (pSTAT3 Tyr705; (**H**) increased. β-actin served as the loading control. (**G**,**H**) Densitometric quantification of the Western blot bands (**F**). (**I**,**J**) Representative immunohistochemistry images for NF-κB localization in mouse skin (scale bar = 50 μm). (**J**) Quantification of nuclear NF-κB translocation from (**I**). Data are expressed as the mean ± SD. *, *p* < 0.05 and **, *p* < 0.01, vs. first bar; $, *p* < 0.05, vs. fourth bar (Mann–Whitney U test). NADPH, nicotinamide adenine dinucleotide phosphate; IL-10, interleukin-10; PN, polynucleotides; TGF-β, tumor growth factor-β.

The NADPH/NADP^+^ ratio was increased by PN treatment and continued to rise over time in the aged skin (Figure 5A).

Western blot analysis of the skin was used to assess M2 polarization by quantifying the expression of CD86 and CD206. The extent of M2 polarization was expressed as a ratio of CD206/(CD86 + CD206). The ratio of CD206/(CD86 + CD206) was increased by PN and the highest expression observed 3 days after PN injection (Figure 5B,C).

PN elevated the levels of IL-10 and TGF-β, and this effect increased over time (Figure 5D,E).

Similarly, the ratios of pSMAD2/3/SMAD2/3 and pSTAT3/STAT3 were increased by PN, and their effects increased over time (Figure 5F–H).

NF-κB nucleus intensity was decreased by PN, and it continued to decrease over time (Figure 5I,J).

### 2.6. PN Increased Collagen Density in the Aged Skin

**Figure 6:**
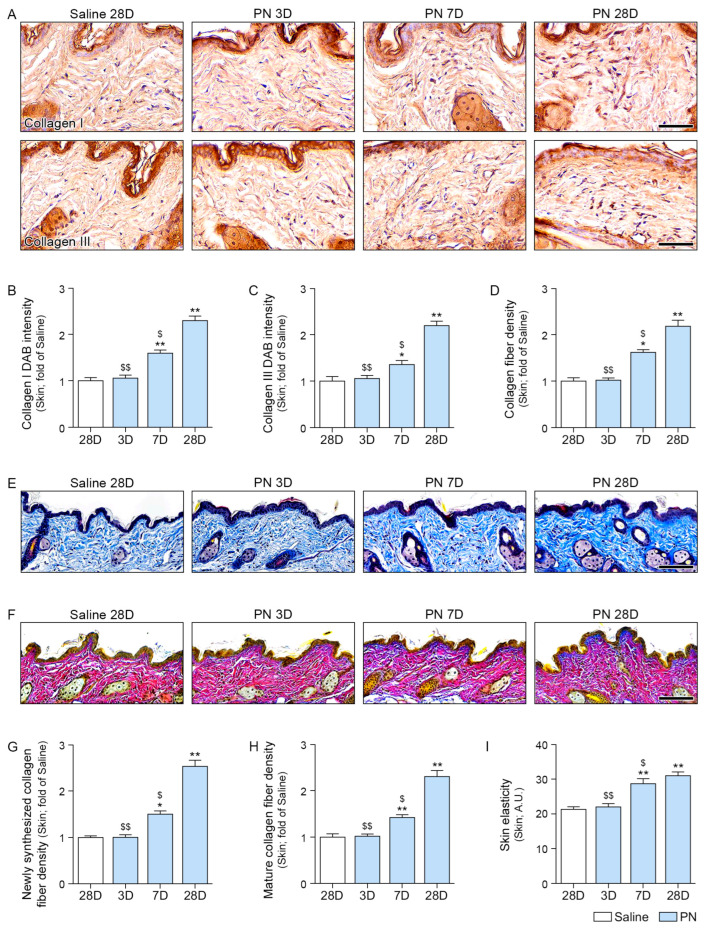
PN enhances collagen synthesis, fiber density, and skin elasticity in mouse skin. (**A**) Representative immunohistochemistry images of collagen I and collagen III expression at 3, 7, and 28 days after PN treatment (scale bar = 50 μm). (**B**,**C**) Quantification of DAB intensity for collagen I (**B**) and collagen III (**C**), respectively. (**D**,**E**) Masson’s trichrome staining for visualization of collagen fibers in the dermis (scale bar = 100 μm). (**F**–**H**) Herovici staining is used to distinguish newly synthesized collagen fibers (scale bar = 100 μm). (**G**,**H**) Quantification of newly synthesized collagen fiber density (**G**) and mature collagen fiber density (**H**). (**I**) Skin elasticity was significantly increased in the PN-treated groups. Data are expressed as the mean ± SD. *, *p* < 0.05 and **, *p* < 0.01, vs. first bar; $, *p* < 0.05 and $$ *p* < 0.01, vs. fourth bar (Mann–Whitney U test). PN, polynucleotides.

Collagen I and III levels were increased by PN treatment, and this effect became more pronounced over time (Figure 6A–C).

Collagen density, evaluated by Masson’s trichrome straining, was also elevated by PN, with the effect increasing over time (Figure 6D,E).

Herovici staining was performed to distinguish between newly formed collagen (blue) and mature collagen (red) [26,27]. Both newly synthesized and mature collagen were increased by, and their levels continued to rise over time (Figure 6F–H).

Skin elasticity was evaluated using an API-100 Skin Analysis Machine (Aram Huvis, Republic of Korea). PN treatment led to an increase in skin elasticity, with the effect becoming more evident over time (Figure 6I).

## 3. Discussion

Both PDRNs and PNs refer to polymers composed of multiple units of deoxyribonucleotides [3]. In the aesthetic and cosmeceutical fields, PDRNs and PNs have been shown to decrease oxidative stress and inflammation, improve skin texture, and reduce wrinkles [23,28].

PDRN and PN have been used interchangeably to describe substances containing DNA fragments, which can lead to confusion when scientifically or medically evaluating their effects [3]. Recent proposals have suggested distinguishing between the two substances based on a molecular weight cutoff of 1500 kDa [3].

A decline in function of fibroblasts is closely associated with reduced collagen synthesis during skin aging and impaired wound healing [29,30]. Beyond fibroblasts, skin regeneration involves complex interactions among various cell types, including keratinocytes and macrophages [30,31]. Therefore, research efforts are aimed at enhancing the proliferation and migration of these cells or reducing inflammation to mitigate the deterioration of their functions. In this context, numerous studies have investigated the use of peptides to increase the migration and proliferation of various skin cells [32,33,34,35]. These peptides typically bind to specific cell surface receptors, activating distinct intracellular signaling pathways that lead to enhanced cell function [32,33,34,35]. Similarly, PDRN are known to bind to adenosine receptors, which subsequently promote fibroblast proliferation and thereby amplifies the wound healing process [1,36].

Although PDRN has long been known to exert its effects by activating adenosine receptors, experimental evidence of PN directly activating adenosine receptors is lacking. In this study, we investigated whether PN activated adenosine receptors, focusing specifically on their activation in macrophages, which can influence fibroblasts.

In our study, A2AR expression in senescent macrophages increased with increasing concentrations of PN. PN also increased the AC and cAMP levels, which were enhanced by A2AR.

Previous research indicates that the cAMP/PKA/CREB pathway can decrease NF-κB, and CREB-activated MAPK can reduce inflammatory responses [6,37]. It is also known that CREB promotes IL-10 secretion, thereby inducing M2 polarization [38]. Furthermore, PCK1 is involved in gluconeogenesis and inhibits ROS production, which can suppress M1 polarization [14]. PCK1 is also known to be activated by CREB [11]. Building on these findings, we conducted experiments to determine whether PN activates the cAMP/PKA/CREB pathway, leading to PCK1 activation, which, in turn, reduces oxidative stress and suppresses M1 polarization, ultimately increasing the M2 polarization ratio.

Our experimental results using senescent macrophages were consistent with our hypotheses. In senescent macrophages, PN increased the levels of PKA/CREB/PCK1 and decreased oxidative stress. M2 ratio and M2 cytokines such as IL-10 and TGF-β were increased by PN. These increases were decreased by the PCK1 inhibitor, suggesting that PN leads to increased M2 macrophages and M2 cytokine secretion via PCK1 upregulation. These results suggest that PN increases collagen synthesis in senescent fibroblasts by modulating macrophage PCK1 activity. This is the most interesting finding of this study. Previous studies showed that PDRN decreased nitric oxide and TNF-α in the murine macrophage cells [9]. Moreover, PDRN increased IL-10 in the human chondrocytes [39]. Thos results have suggested that the anti-inflammatory effects of PDRN and PN may involve reducing M1 polarization or increasing M2 polarization. However, direct experimental evidence demonstrating how they induce M1 or M2 polarization is lacking. The current study showed that the ability of PN to increase M2 polarization was diminished by a PCK1 inhibitor. To our knowledge, this is the first study to identify PCK1 as a potential link between the A2AR/AC/cAMP/PKA/CREB pathway and M2 polarization.

Macrophages are the first cells to respond to implanted biomaterials [40]. Fibroblasts are primarily responsible for collagen synthesis in the skin; thus, the interaction between macrophages and fibroblasts is crucial for biomaterial-induced collagen production [41]. Notably, IL-10 and TGF-β are key cytokines secreted by macrophages that promote collagen synthesis in fibroblasts [41,42].

Our results showed that CM from PN-treated macrophages increased pSMAD2/3 and pSTAT3, leading to increased collagen and decreased NF-κB activity in the senescent fibroblast. Conversely, treatment with CM from PCK1 inhibitor-treated macrophages resulted in the opposite pattern of change compared with treatment with CM from PN-treated macrophages. These results suggest that PN increases collagen synthesis in senescent fibroblasts via macrophage PCK1 modulation. Previous studies have shown that during the wound healing process, activated macrophages stimulate fibroblasts, and this effect is primarily attributed to key macrophage-secreted factors such as IL-10 and TGF-β [41,42]. However, few studies have investigated whether changes in macrophage PCK1 can also influence fibroblasts. Our findings demonstrate that PCK1 plays a role in the intricate macrophage-fibroblast crosstalk, contributing to the collagen synthesis process.

Our in vivo results are consistent with our in vitro findings. The expression of the A2AR/AC/cAMP/PKA/CREB/PCK1 pathway was the highest 3 days after PN injection into the skin. However, the oxidative stress-reducing effect and the expression of IL-10 and TGF-β showed a tendency to increase over time, peaking at 28 days post-injection. Collagen density peaked after 28 days. These results suggest that while PN rapidly increased PCK1 expression shortly after injection, the anti-inflammatory and oxidative stress-reducing effects mediated by PCK1 persisted for up to 28 days, leading to a sustained increase in collagen synthesis. It has been reported that the half-life of PDRN is 3 h when injected into mice peritoneum [1]. However, the biological effect of PDRN is more prolonged than its half-life, as nucleotides are the main factors that show biological effects [1]. Pharmacokinetic studies of PN are limited, and its precise half-life has not been definitively reported. However, based on the principle that high-molecular-weight substances generally undergo slower enzymatic degradation compared to their low-molecular-weight counterparts, it can be hypothesized that PN degrades more slowly than PDRN. This slower degradation may allow PN to remain in the skin for a longer duration, thereby exerting beneficial effects over an extended period.

The present study elucidates the foundational role of the PCK1 pathway in PN-mediated collagen synthesis; however, certain limitations must be recognized. First, while our experimental model focused on the paracrine effects of macrophages on fibroblasts, we cannot exclude the possibility of direct PN action on fibroblasts. Fibroblasts are known to express A2AR, and its activation can directly enhance collagen synthesis via the AKT pathway [43]. Therefore, future research should explore the direct effects of PN on fibroblasts independent of macrophage involvement to fully characterize its mechanism of action. Second, our results are confined to a senescent cell model. Given that PCK1 function itself changes with aging [44], it remains uncertain whether PN would exert a similar effect in healthy, non-senescent cells. Therefore, future studies are warranted to investigate whether PN similarly enhances collagen synthesis and skin regeneration by modulating PCK1 activity in normal, non-senescent models. Third, our study focused on the effect of PN-induced PCK1 on macrophage polarization. However, previous research indicates that PCK1 activity is a central regulator of cellular metabolism, influencing protein synthesis, lipogenesis, gluconeogenesis, and mitochondrial function, all of which are closely linked to aging [45,46]. Therefore, while we have established a link between the PN-PCK1 axis and macrophage polarization, it remains a question whether the observed PN-mediated enhancement of collagen synthesis is also influenced by alterations in protein synthesis or mitochondrial metabolism via the PCK1 pathway. Further investigation into these broader metabolic effects of PN is warranted. Finally, as the primary objective of this study was to investigate the molecular mechanism by which PN regulates PCK1 to enhance collagen synthesis, we did not perform pharmacokinetic investigations or assess the dermal residence time of PN. These aspects are crucial for accurately evaluating the biological safety and efficacy of PN in a clinical setting. Future studies addressing these factors are necessary for a more comprehensive understanding of PN’s therapeutic potential.

Despite these limitations, our findings demonstrate that PN stimulates adenosine receptors in macrophages, which are the primary cells encountered upon PN administration. This stimulation led to increased PCK1 expression and subsequently enhanced M2 polarization and promoted collagen synthesis in fibroblasts. This study provides the first direct experimental evidence that PCK1 acts as a downstream mediator of adenosine receptor activation, driving M2 polarization, which was previously missing from our understanding of how PN-mediated adenosine receptor activation concretely reduces inflammation in macrophages.

## 4. Materials and Methods

### 4.1. PN Preparation

The sodium polynucleotides used in this study was provided by RF Bio Co. Ltd. (Gangwon, Republic of Korea). Sodium polynucleotides, the main component, was extracted from homogenized salmon testis tissue. The homogenate was adjusted to pH 5.5 to facilitate enzymatic protein degradation, followed by filtration. The nucleic acids were precipitated and purified. The final product had a molecular size of 400 ± 50 base pairs, with a 260/280 absorbance ratio of 1.9, indicating high purity. The complete PN formulation consisted of sodium polynucleotides (25 mg/mL), hyaluronic acid (10 mg/mL), lidocaine hydrochloride (3 mg/mL), glycerin (5 mg/mL), and glutathione (1 mg/mL) [47].

Each production batch was subjected to comprehensive quality control procedures, including pH measurement, loss on drying, protein content, PN content, heavy metal analysis, residual solvent testing, microbial limit tests, and PN identity confirmation. These tests were conducted in accordance with the manufacturer’s internal certificate of analysis (CoA, available upon request).

### 4.2. In Vitro Experiments

#### 4.2.1. Cell Culture

THP-1 human monocytes (Korea Cell Line Bank, Seoul, Republic of Korea) were cultured in RPMI-1640 medium (Welgene, Gyeongsan, Republic of Korea) supplemented with 10% fetal bovine serum (FBS; Gibco, Waltham, MA, USA) and 1% penicillin–streptomycin (Welgene). To macrophage differentiation, the cells were treated with 100 ng/mL of 4β-phorbol-12-myristate-13-acetate (PMA; Sigma-Aldrich, St. Louis, MO, USA) and incubated for 24 h prior to use in subsequent experiments [48]. HDF human dermal fibroblasts (Cefobio, Seoul, Republic of Korea) were maintained in CEFO^TM^ Human Dermal Fibroblast cells kit (Cefobio). All cells were maintained in a humidified environment with 5% CO_2_ at 37 °C, and cells were used in the experiments when the cells reached approximately 80% confluence.

#### 4.2.2. Experimental Design for PN Treatment

To investigate the effects of PN on macrophage senescence, differentiated macrophages were cultured for 24 h, treated with 100 μM H_2_O_2_ for 3 h, and cultured for 72 h to generate senescent cells (Figure S1). These senescent macrophages were subsequently exposed to PN at concentrations ranging from 0 to 20 mg/mL for an additional 48 h to determine cytotoxicity. After testing at 0–3 mg/mL, 2 mg/mL was identified as the optimal concentration for further cell experiments. For the main experiments, proteins were extracted from cell lysates, and the macrophage culture medium was collected for HDF culture.

#### 4.2.3. PCK1 Inhibition

To determine whether the PN-mediated effects depend on PCK1 activity, macrophages were chemically inhibited using 3-mercaptopicolinic acid (3-MPA). Differentiated macrophages were cultured for 24 h, treated with 100 μM H_2_O_2_ for 3 h, and cultured for 72 h to generate senescent cells. Cells were pretreated with 0.25 mM 3-MPA or PBS (vehicle control) for 24 h [49,50]. Then, 2 mg/mL PN or PBS were replaced, and the cells were cultured for an additional 48 h. Cell lysates and supernatants were collected for subsequent analyses.

To investigate the effects of PN on fibroblast senescence, fibroblasts were cultured for 24 h, treated with 350 μM H_2_O_2_ for 3 h, and cultured for 72 h to generate senescent cells (Figure S3). Then, CM from senescent macrophages were replaced, and the cells were cultured for an additional 48 h. Cell lysates were collected for subsequent analyses.

### 4.3. Cell Viability Assessment

To evaluate PN cytotoxicity, senescent macrophages were treated with increasing concentrations of PN (0–20 mg/mL) for 48 h. After treatment, CCK-8 reagent (TransGen Biotech Co., Ltd., Beijing, China) was added to each well, and the optical density was measured at 450 nm after 2 h of incubation.

### 4.4. Quantitative Reverse Transcription Polymerase Chain Reaction (RT-qPCR)

RNA was isolated from the cells using RNAiso reagent (TAKARA, Tokyo, Japan). The yield and RNA purity were evaluated with a Nanodrop spectrophotometer (Thermo Fisher Scientific, Waltham, MA, USA), and 1 μg of RNA was used to reverse-transcribed following the manufacturer’s protocol.

RT-qPCR was carried out a QuantStudio™ 3 Real-Time PCR System (Thermo Fisher Scientific) using SYBR green premix (TAKARA). Gene expression levels were determined with ΔΔCT method and normalized.

### 4.5. Flow Cytometry

The macrophages were resuspended in PBS containing 1% FBS (fluorescence-activated cell sorting buffer) at a density of 1 × 10^5^ cells/mL. The macrophages were then incubated with anti-CD86, anti-CD206, and Alexa Fluor^®^ 488, and Alexa Fluor^®^ 594 antibody in FACS buffer for 30 min at 4 °C in the dark. The stained cells were analyzed using a flow cytometer (BD FACS Calibur; BD Biosciences, Franklin Lakes, NJ, USA) at Core-facility for Cell to In-vivo im-aging. Data was collected and analyzed using BD FACSDiva 9.0 (BD Biosciences, Franklin Lakes, NJ, USA). Data obtained from flow cytometry were expressed as mean fluorescence intensity and the positive cells.

### 4.6. In Vivo Experiments

#### 4.6.1. Mouse Model and Maintenance

Six-week-old C57BL/6N mice were purchased from Orient Bio Inc. (Seongnam, Republic of Korea). After mating, the offspring were allowed to age naturally. At 17 months of age, male animals were randomly divided into four groups for experimental treatment (*n* = 5 per group) [51].

During the study, the mice were housed in a controlled environment with a constant temperature of 20–24 °C and humidity of 45–55%, with free access to standard laboratory food and water. All animal experimental protocols were approved by the Institutional Animal Care and Use Committee (IACUC) of Gachon University (Approval Number: LCDI-2023-0095). Experimental procedures were performed in accordance with the national and institutional guidelines for ethical animal experimentation.

#### 4.6.2. Experimental Design for PN Injections

Group 1 was injected with sterile saline and served as the control. Groups 2–4 received injection of 200 μL of PN into a 2 cm × 2 cm area of dorsal tissue using a 27-gauge needle. The injection was performed evenly so that the solution was distributed to multiple sites (4 sites × 50 μL). Skin tissues were harvested from the injection site at different time points (3, 7, or 28 days) after administration. Skin samples were excised, fixed in 4% paraformaldehyde for histological analysis, or rapidly frozen for molecular analysis.

#### 4.6.3. Skin Elasticity

Skin elasticity was assessed using an API-100 device (Aram Huvis, Seongnam, Republic of Korea). High-resolution images of the skin surface are captured using a non-contact optical method, and elasticity is quantitatively analyzed using the accompanying software. All animals were measured five times immediately prior to sampling, and the average value was calculated.

### 4.7. Sample Preparation

#### 4.7.1. Protein Isolation and Concentration Quantitation

Proteins were extracted using EzRIPA buffer (ATTO Corporation, Tokyo, Japan), and concentrations were then determined with a bicinchoninic acid (BCA) assay kit (Thermo Fisher Scientific, Waltham, MA, USA). All procedures followed the guidelines provided by the suppliers of each reagent.

#### 4.7.2. Paraffin-Embedded Skin Tissue Blocks

Tissue was fixed in 4% paraformaldehyde (Sigma-Aldrich, St. Louis, MO, USA) for 72 h, processed automated tissue processor (Leica, Wetzlar, Germany), and subsequently dehydrated and embedded in paraffin. The tissue was cut into 7 µm using a microtome and transferred onto slides, followed by incubation at 60 °C overnight.

### 4.8. Enzyme-Linked Immunosorbent Assay

Microplates were first coated with a capture antibody in carbonate–bicarbonate buffer (pH 9.6, sodium counterion) and then blocked with 5% skim milk. Protein was loaded with wells, washed protein, followed incubated primary antibodies overnight at 4 °C (diluted in PBS; Table S1). After additional washing, peroxidase-conjugated secondary antibody (1:10,000; Vector Laboratories, Newark, CA, USA) was applied at room temperature for 3 h. To detect protein, a tetramethylbenzidine substrate (Sigma-Aldrich, St. Louis, MO, USA) was added and allowed, and stopped with 1 M sulfuric acid (Sigma-Aldrich). The absorbance recorded at 450 nm using a microplate reader (Thermo Fisher Scientific).

Assay for adenylate cyclase (Mybiosource, Inc., Vancouver, BC, Canada), cyclic AMP concentration (Cell Signaling Technology, Inc., Danvers, MA, USA), and NADPH/NADP^+^ (Abcam Limited, Cambridge, UK) followed the guidelines provided by the suppliers of each reagent.

### 4.9. Western Blotting

Equal amount of protein from cell lysates or skin samples were separated using sodium dodecyl sulfate polyacrylamide gel electrophoresis, transferred to a 45 µm polyvinylidene fluoride membrane, and blocked in 5% skim milk. After three washes, the membrane was incubated overnight at 4 °C with the primary antibody (Table S1). After further washing, peroxidase-conjugated secondary antibody (1:10,000; Vector Laboratories) was incubated at room temperature for 1 h. Protein bands were visualized using chemiluminescent solutions and captured using a ChemiDoc MP Imaging System (Bio-Rad, Hercules, CA, USA), and for image acquisition, we used Image Lab software version 6.1 (Bio-Rad, Hercules, CA, USA).

### 4.10. Staining

#### 4.10.1. Immunocytochemistry

The fibroblasts with conditioned media (CM) from senescent macrophages were washed PBS, blocked via incubation with a serum solution for 1 h at room temperature, and incubated the slides with a primary antibody overnight at 4 °C (Table S1). After further washing, Alexa Fluor^®^ 488 conjugated secondary antibody (Invitrogen) was incubated at room temperature for 1 h. The cells were counterstained with DAPI (Sigma-Aldrich, St. Louis, MO, USA) for 30 sec and mounted in glycerol mounting solution (Vector Laboratories, Newark, CA, USA). The stained cells were visualized using an LSM-710 microscope.

#### 4.10.2. Immunohistochemistry

Paraffin-embedded sections were deparaffinized, blocked via incubation with a serum solution for 1 h at room temperature, and incubated the slides with a primary antibody overnight at 4 °C (Table S1). After PBS washes, the slides were incubated with a biotinylated secondary antibody (Vector Laboratories) for 1 h at room temperature, followed by further rinsing with PBS. The slides were then treated with ABC reagent (Vector Laboratories) and washed. The 3,3′-diaminobenzidine (Sigma-Aldrich) substrate revealed positive staining as brown, and counterstained with hematoxylin (KPNT, Cheongju, Republic of Korea). After staining, the slides were sealed with DPX mounting solution (Sigma-Aldrich), scanned using a slide scanner (Motic Scan Infinity 100; Motic, Beijing, China), and images were captured.

#### 4.10.3. Masson Trichrome Staining

To stain collagen fibers in skin tissue, masson trichrome (Scytek Laboratories, West Logan, UT, USA) stain were performed according to the manufacturer’s instructions. After staining, the slides were sealed with DPX mounting solution (Sigma-Aldrich, St. Louis, MO, USA), scanned using a slide scanner (Motic Scan Infinity 100; Motic, Beijing, China), and images were captured. In this staining method, collagen fibers appear blue [52].

#### 4.10.4. Herovici Staining

To differentiate new synthesized collagen fibers from mature fibers, herovici (Scytek Laboratories, Logan, UT, USA) stain followed the guidelines provided by the suppliers of each reagent. After staining, the slides were visualized using a slide scanner, as with other stains [26,27].

### 4.11. Quantitative and Statistical Analysis

Quantification was carried out with ImageJ software version 1.53s (NIH). For each parameter, experimental groups were compared with the control [53,54]. Results are shown as mean ± standard deviation. Differences among groups were analyzed using the Kruskal–Wallis and post hoc pairwise comparisons were conducted with the Mann–Whitney U. Statistical significance is noted in each figure legend. All statistical analyses were performed using SPSS version 26 (IBM Corp., Armonk, NY, USA).

## 5. Conclusions

In summary, this study elucidated a novel mechanism by which PN enhances collagen synthesis in aged skin, primarily through the upregulation of PCK1 in senescent macrophages and subsequent modulation of the macrophage-fibroblast axis. PN was shown to activate the A2AR/AC/PKA/CREB/PCK1 signaling cascade, leading to reduced oxidative stress, increased M2 macrophage polarization, and elevated secretion of IL-10 and TGF-β. These cytokines, in turn, activated SMAD2 and STAT3 in fibroblasts, promoting collagen synthesis and inhibiting NF-κB-mediated collagen degradation. These in vivo findings corroborated the in vitro results, demonstrating sustained improvements in collagen density and skin elasticity following PN administration.

Our findings suggest PN may serve as an effective agent for skin rejuvenation therapies, particularly in aging populations, where collagen loss and chronic inflammation contribute to skin deterioration. By targeting the key pathways involved in cellular senescence and extracellular matrix remodeling, PN offers a promising approach for restoring skin structure and function. However, while our preclinical data is encouraging, more research is needed to further confirm their efficacy and optimization for human applications. Therefore, large-scale randomized controlled trials are warranted to validate the long-term safety, optimal dosing, and clinical outcomes of PN in human subjects

## Data Availability

All data are contained within the article.

## Supplementary Materials

The following supporting information can be downloaded at: https://www.mdpi.com/article/10.3390/ijms26178720/s1.
