# Supplementary material for: Polynucleotides Enhance Collagen Synthesis via Modulating Phosphoenolpyruvate Carboxykinase 1 in Senescent Macrophages: Experimental Evidence
Source: Int J Mol Sci. 2025 Sep 7;26(17):8720. doi: 10.3390/ijms26178720 (PMC12429772; doi:10.3390/ijms26178720)
Supplement: Supplementary file 1 [file ijms-26-08720-s001.zip › ijms-3843907-supplementary.pdf]

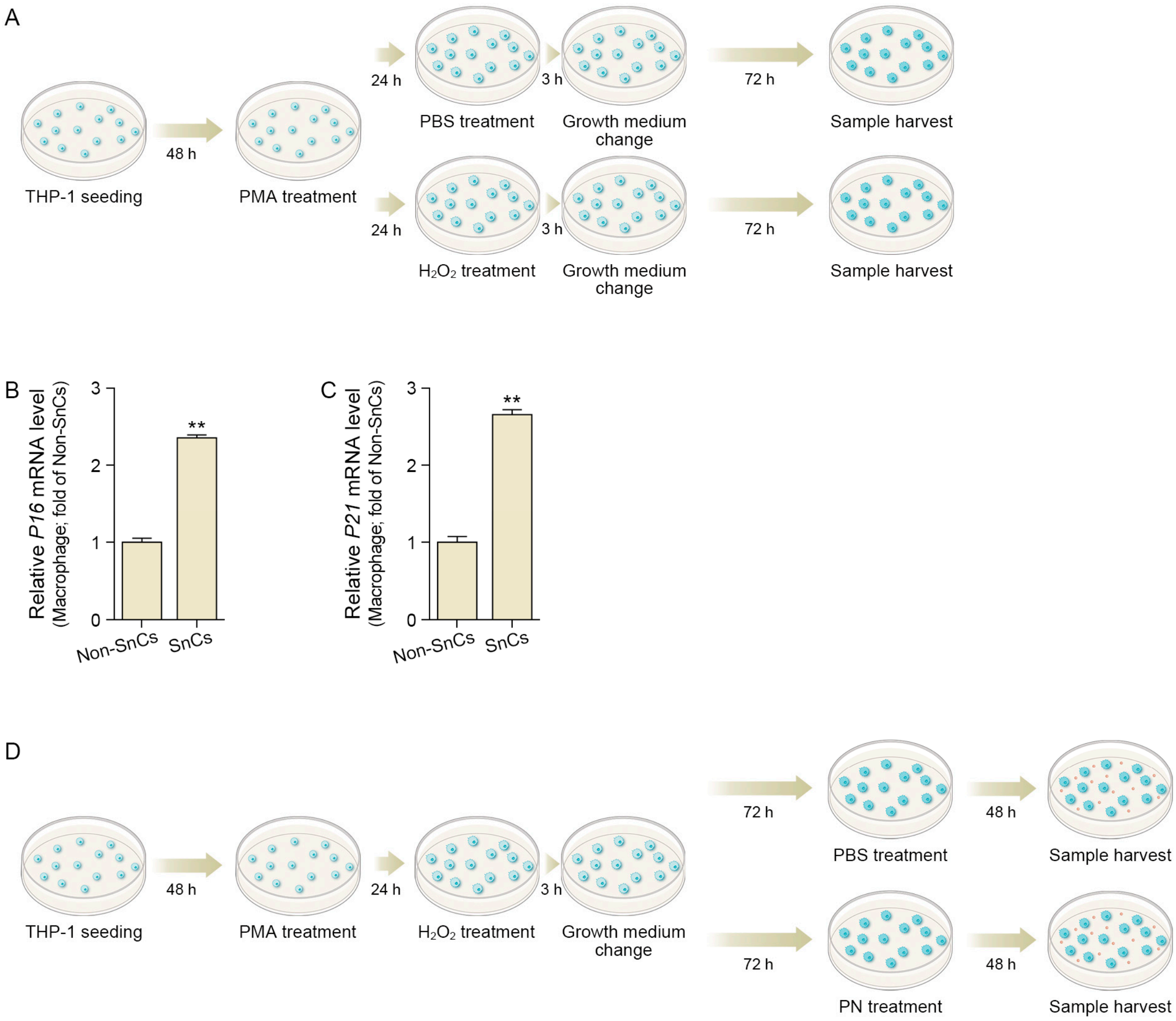

Figure S1. Confirmation of macrophage senescence and evaluation of PN efficacy in senescent macrophages. (A) Cell schematic to confirm macrophage senescence. (B,C) Quantitative PCR analysis of *P16* (B) and *P21* (C) expression in macrophages. (D) Cell schematics to confirm the efficacy of PN in senescent macrophages. Data are expressed as the mean ± SD. **, $p < 0.01$, vs. first bar (Kruskal–Wallis test). PBS, phosphate-buffered saline; PCK1, phosphoenolpyruvate carboxykinase 1; PCR, polymerase chain reaction; PN, polynucleotides.

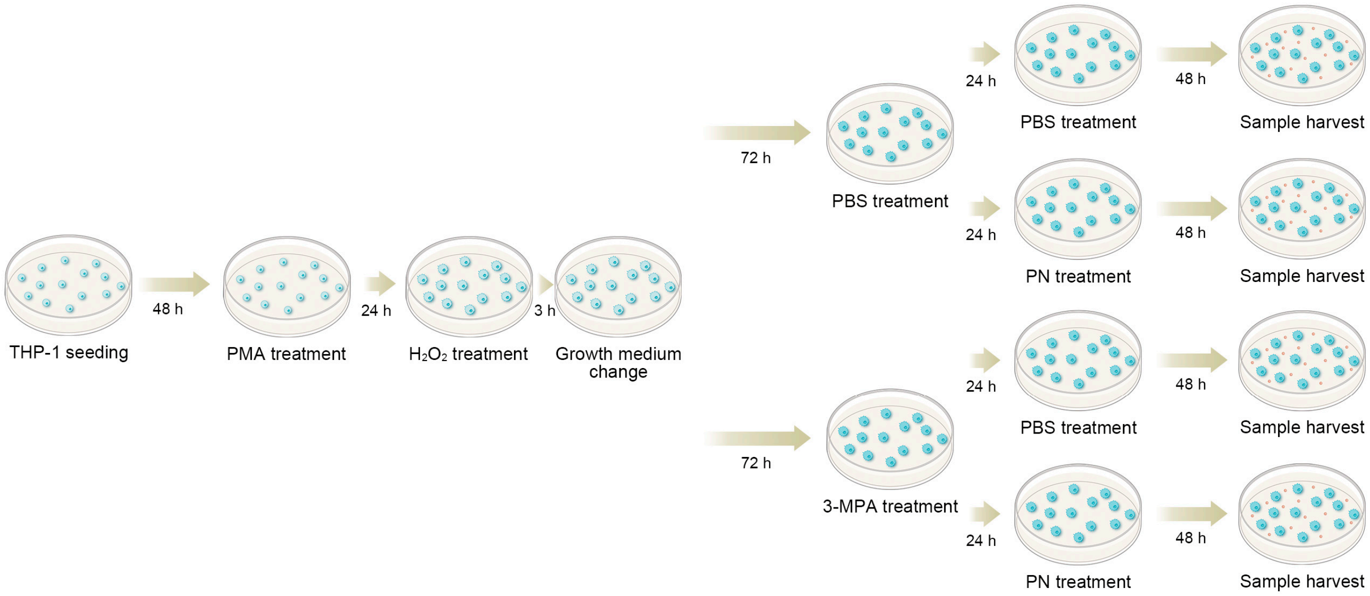

Figure S2. Cell schematics to confirm the efficacy of PN-mediated effects depend on PCK1 in senescent macrophages.

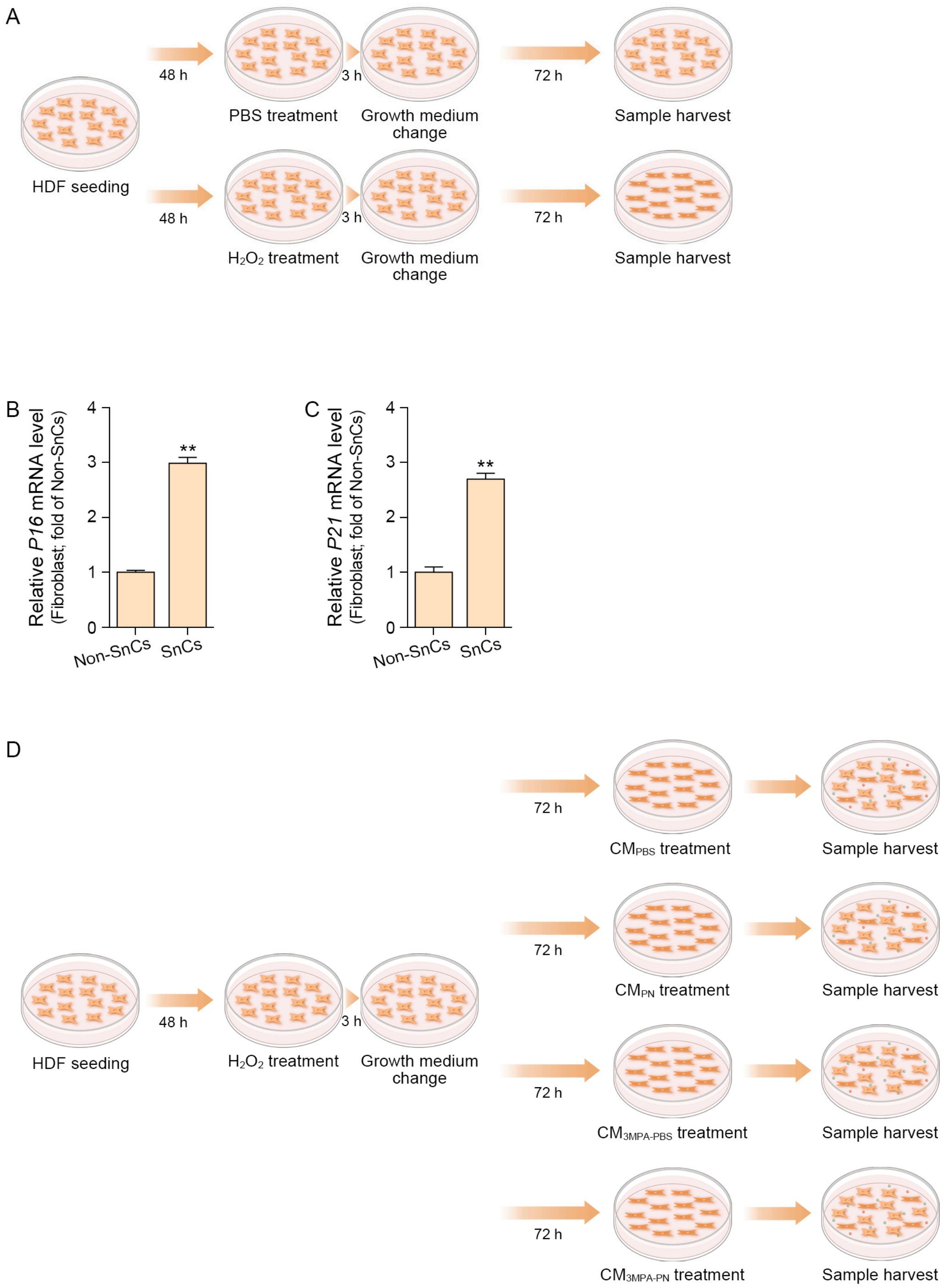

Figure S3. Confirmation of fibroblast senescence and evaluation of PN efficacy in senescent fibroblast. (A) Cell schematic to confirm fibroblast senescence. (B,C) Quantitative PCR analysis of *P16* (B) and *P21* (C) expression in fibroblasts. (D) Cell schematics to confirm the efficacy of PN-mediated effects depend on PCK1 in senescent fibroblasts. Data are expressed as the mean ± SD. **, $p < 0.01$, vs. first bar (Kruskal–Wallis test). CM, conditioned media; PBS, phosphate-buffered saline; PCK1, phosphoenolpyruvate carboxykinase 1; PCR, polymerase chain reaction; PN, polynucleotides.

Table S1. List of antibodies for enzyme-linked immunosorbent assay (ELISA), western blot (WB) and immunocytochemistry (ICC) / immunohistochemistry (IHC).

Antibody	Company	Dilution Rate		
		ELISA	WB	ICC/IHC
PKA	Santa cruz	-	1:500	-
CREB	Sigma-Aldrich	-	1:1,000	-
pCREB	Cell signaling	-	1:1,000	-
PCK1	Cell signaling	-	1:1,000	-
β-actin	Cell signaling	-	1:1,000	-
CD86	Santa cruz	-	1:1,000	-
CD206	Santa cruz	-	1:1,000	-
IL-10	Fine Test	1:500	-	-
TGF-β	Stressmarq	1:200	-	-
SMAD2/3	Cell signaling	-	1:1,000	-
pSMAD2/3	Cell signaling	-	1:1,000	-
STAT3	Cell signaling	-	1:1,000	-
pSTAT3	HUABIO	-	1:1,000	-
NF-κB	Cell signaling	-	-	1:200
Collagen I	Santa cruz	-	-	1:100
Collagen III	Abcam	-	-	1:100
